# mRNA export in the apicomplexan parasite *Toxoplasma gondii*: emerging divergent components of a crucial pathway

**DOI:** 10.1186/s13071-018-2648-4

**Published:** 2018-01-25

**Authors:** Andréa Rodrigues Ávila, Alexjandro Cabezas-Cruz, Mathieu Gissot

**Affiliations:** 1 Instituto Carlos Chagas, FIOCRUZ, Rua Algacyr Munhoz Mader, 3775. CIC, Curitiba, PR 81350-010 Brazil; 2 UMR BIPAR, Animal Health Laboratory, ANSES, INRA, ENVA, Maisons Alfort, Cedex France; 3 Institute of Parasitology, Biology Center, Czech Academy of Sciences, České Budějovice, Czech Republic; 4 Faculty of Science, University of South Bohemia, České Budějovice, Czech Republic; 5 University of Lille, CNRS, Inserm, CHU Lille, Institut Pasteur de Lille, U1019 - UMR 8204 - CIIL - Centre d’Infection et d’Immunité de Lille, F-59000 Lille, France

**Keywords:** mRNA export, Parasites, Apicomplexa, Gene expression, *Plasmodium*, *Cryptosporidium*, *Toxoplasma*

## Abstract

Control of gene expression is crucial for parasite survival and is the result of a series of processes that are regulated to permit fine-tuning of gene expression in response to biological changes during the life-cycle of apicomplexan parasites. Control of mRNA nuclear export is a key process in eukaryotic cells but is poorly understood in apicomplexan parasites. Here, we review recent knowledge regarding this process with an emphasis on *T. gondii*. We describe the presence of divergent orthologs and discuss structural and functional differences in export factors between apicomplexans and other eukaryotic lineages. Undoubtedly, the use of the CRISPR/Cas9 system in high throughput screenings associated with the discovery of mRNA nuclear export complexes by proteomic analysis will contribute to identify these divergent factors. Ligand-based or structure-based strategies may be applied to investigate the potential use of these proteins as targets for new antiprotozoal agents.

**Electronic supplementary material:**

The online version of this article (10.1186/s13071-018-2648-4) contains supplementary material, which is available to authorized users.

## Background

Apicomplexan parasites are the infectious agents of severe illnesses of medical and veterinary importance. These parasites comprise a wide range of protozoans, among which *Plasmodium falciparum* and *Toxoplasma gondii* represent serious threats to human health. With no vaccine currently available, the discovery of new drugs is urgently needed to treat these diseases. For *Plasmodium* spp. in particular, widespread resistance to commonly employed therapies is a subject of concern. Advances in understanding the cell biology of infectious agents will contribute to discover new control strategies.

Control of gene expression during the life-cycle of parasites is crucial for their pathogenicity, differentiation, immune evasion and drug resistance [1]. Gene expression can be regulated by diverse processes, affecting mRNA at the level of transcription, processing, nuclear export, translation and also at the level of protein stability. At each step, these processes can be regulated to allow fine-tuning of gene expression. Regulation mechanisms such as epigenetic, transcription or translation pathways have been showed to play important regulatory roles for apicomplexan parasites [2–7]. Transcription produces precursor mRNAs (pre-mRNAs) that are processed, folded, and assembled into RNA-protein (RNP) complexes. Integrated nuclear processes are involved in the formation and export of mRNA-protein complexes (mRNPs), and specific proteins are essential for export and control of the cytoplasmic fate of mRNAs [8]. In mammals, mRNA export can be selective for regulating crucial biological processes [9], and nuclear transport of mRNA is key to gene expression regulation in eukaryotic cells. Recently, the control of mRNA export was shown to contribute to stage-specific gene expression control in *Trypanosoma cruzi* [10], a protozoan parasite. However, this process in these parasites remains poorly understood. In the case of early divergent eukaryotic supergroups, such as Chromalveolata and Excavata, few conserved factors have been identified, suggesting the presence of a divergent pathway [11, 12]. Differences in the proteins and pathways controlling gene expression between parasites and the human host may be exploited to discover potential drug targets for anti-parasite chemotherapy.

Protozoan parasites retained canonical proteins associated to processes such as transcription and translation; however, they also employ specific proteins and mechanisms to carry out these processes [1]. Unique actors such as ApiAP2 transcription factors have already been described in apicomplexan parasites [13]. Regarding mRNA export, recent evidence strongly suggests divergent mechanisms in trypanosome parasites [14, 15]; along the same lines, the mRNA export pathway might also be divergent in apicomplexan parasites. Here, we review the data available, mainly for *T. gondii*, describing a general lack of conservation among proteins involved in mRNA export. It may suggest that certain divergent orthologs can fulfill key functions in apicomplexan parasites. Although most of the current knowledge on this pathway in these parasites is based on experiments performed with *T. gondii*, bioinformatic analysis suggests the conservation of mRNA export components in different species of the Apicomplexa (Additional file 1: Table S1). As *T. gondii* is a good model for studying the biology of the Apicomplexa [16], dissection of the macromolecular complexes involved in mRNA export in this parasite can provide a better understanding of this process in other apicomplexan parasites.

## Conserved aspects of nucleocytoplasmic transport in Metazoa and Fungi

### Ran-GTP-dependent pathways

To ensure an efficient macromolecule exchange between the nucleus and the cytoplasm of eukaryotic cells, the former is perforated by large protein structures termed nuclear pore complexes (NPCs) that allow controlled bidirectional nucleocytoplasmic transport of macromolecules through the double membrane of the nuclear envelope [17]. The organization and composition of NPCs is conserved throughout evolution. Each NPC is composed of multiple copies of approximately 30 different proteins known as nucleoporins (NUPs) [18]. Many NUPs contain repeats of the amino acids phenylalanine (F) and glycine (G) (FG repeats). These FG repeats form the central channel of the pore, by which macromolecules are transported in and out of the nucleus [19]. FG nucleoporins form a physical barrier to prevent the movement of macromolecules larger than 40 kDa [19]; thus, specific transport receptors are required for the transport of large macromolecules (greater than 40 kDa) [20]. Protein nucleo-cytoplasmic transport is based on the presence of specific receptors of the karyopherin-β family that interact with FG NUPs in the central channel of the NPC [19]. The nucleoside state of the Ran protein is also instrumental in the binding and release of the cargo-receptor complex after its passage through the nuclear pore [21]. Inside the nucleus, Ran is associated with GTP via activity of the Ran guanine nucleotide exchange factor regulator of chromosome condensation 1 (RCC1) [22]; in the cytoplasm, RanGAP proteins mediate the hydrolysis of Ran-GTP to Ran-GDP. During nuclear protein import, cargo is bound by karyopherins (such as importin beta), and its release after passage through the pores is mediated by the binding of karyopherins to RanGTP [22]. During nuclear protein export, the release of cargo from the receptor (such as exportin XPO1) into the cytoplasm is mediated by GTP hydrolysis [22]. In metazoans, numerous karyopherins are employed for specific import or export of proteins as well as RNA [23].

The nuclear export of most RNA species, such as micro-RNAs (miRNAs), rRNAs, small nuclear RNAs (snRNAs), and tRNAs, follows the RanGTP-exportin model of transport, with specific exportins involved in different export pathways [24, 25]. Exportin-5 (XPO5, Msn5 in yeast) participates in the export of miRNA precursors, whereas Exportin-t (Los1 in yeast) is involved in tRNA export [26]; both exportins bind directly to RNAs. Exportin-1 (XPO1, CRM1 in yeast) is the transport receptor responsible for carrying snRNAs to the cytoplasm [27, 28], and it is also involved in the export of rRNA and requires the adaptor protein NMD3 to couple rRNA synthetic and processing engines [29–33]. Although nuclear RNA export involves components of Ran-GTP dependent pathways, export of the bulk of mRNA is independent of Ran-GTP and exportins, as described below.

### Nuclear mRNA export: a Ran-GTP-independent pathway

Exportin-1 (CRM-1) is also implicated in the mRNA export pathway and relies on the Ran-GTPAse system to mediate export of subsets of mRNAs [34–36], though the CRM1 pathway is not the major route [37, 38]. Indeed, the majority of mRNAs utilize the specific export receptor TAP/p15 (NXF1/NXT1, mex67/mtr2 in yeast) and other specific components that do not depend on Ran-GTPase [39, 40].

**Fig. 1:**
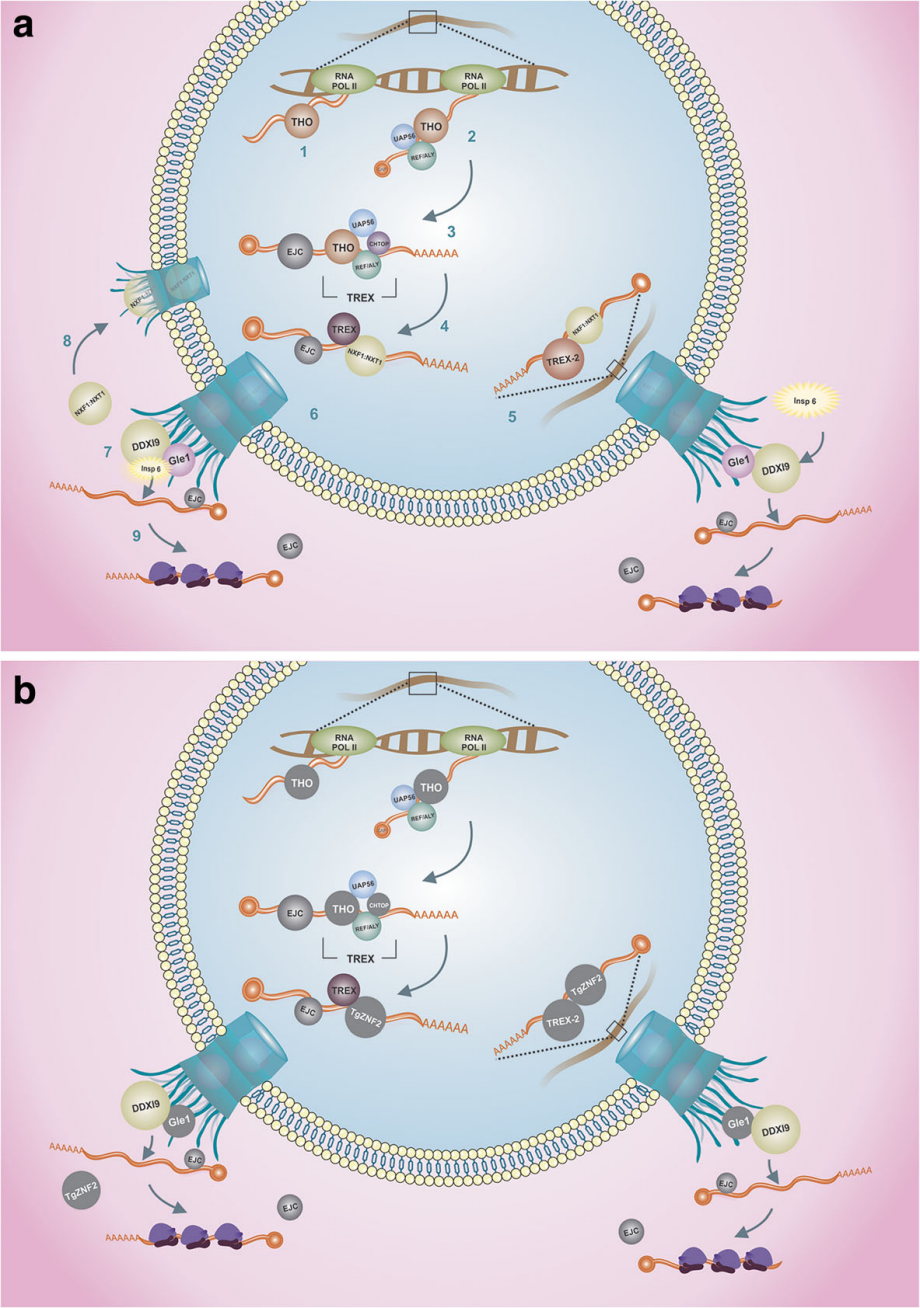
Overview of components in the mRNA export pathway in mammals and *Toxoplasma gondii*. **a** mRNA export in mammalian cells. During gene expression, THO is recruited co-transcriptionally (1), followed by recruitment of the adaptors UAP56 and REF/Aly, which trigger TREX assembly and deposition along the transcript (2). Components of the exon junction complex (EJC) are also bound to mRNA during splicing (3). Once bound to mRNA, TREX recruits the NXF1:NXT1 heterodimer, causing a conformation change in NXF1 and allowing exposure of its RNA-binding domain for interaction with mRNA. This process is promoted by the co-adaptor CHTOP and the adaptor REF/Aly (4). TREX-2 is another complex that interacts with NXF1 and nucleoporins to dock mRNA ribonucleoprotein (mRNP) to the nuclear pore (5). Both TREX and TREX-2 pathways promote mRNP export via NXF1:NXT1. The adaptors are released from mRNP during passage through the nuclear pore complex (NPC) (6). On the cytoplasmic side of the NPC, DDX19 is activated by Gle1 and Isnp6 to trigger the release of NXF1 from mRNA (7), and some components are recycled back to the nucleus (8). The EJC is dissociated from mRNA when translation is initiated (9). **b** Overview of conserved components identified in *Toxoplasma gondii* by bioinformatic searches*.* Conserved components are colored; non-conserved components are shown in gray. A divergent REF/Aly homolog has been identified [75], and TgZNF2 is proposed to be a functional homolog of the export receptor [77]

The formation of export-competent-mRNPs involves recruitment of several export factors that link transcription with mRNA export, as depicted schematically in Fig. 1a. The THO complex associates with the transcriptional machinery during RNA polymerase II (RNA pol II) elongation [41–43]. This complex is composed of Tho2, Hpr1, Mft1 and Thp2 in yeast, and it is conserved in *Drosophila* and humans [44, 45]. The THO complex functions in the cotranscriptional loading of Sub2 and Yra1 onto nascent transcripts to form the TREX (TRanscriptional EXport) complex, coupling transcription with messenger mRNA export [45]. Rather than by the direct transcription-coupled mechanism that occurs in yeasts and in humans, recruitment of the TREX complex, containing REF/Aly (Yra1 in yeast), UAP56 (Sub2 in yeast) and the human counterpart of the THO complex that specifically associates with spliced mRNA, to mRNA occurs via a splicing-coupled mechanism [46, 47].

By interacting with the cellular adaptor protein REF/Aly, UAP56 fulfills a key function for cellular mRNA export [48–50]. REF/Aly was originally described as a component of Exon Junction Complex (EJC), which targets spliced mRNA for export [51]. EJC acts as a platform for transient binding of numerous proteins that define the fate of individual mRNAs for specific maturation pathways [52, 53]. Once REF/Aly is loaded onto mRNA, it recruits TAP (NXF1, mex67 in yeast), which forms a heterodimer with p15 (NXT1, Mtr2p in yeast) [54–56]. Formation of a ternary complex with TAP-p15 displaces UAP56 and causes the transfer of RNA to the export receptor [55]. Another component of TREX, Chtop, is required for remodeling of the export receptor and consequent RNA binding to TAP/NXF1 [56]. The conformation assumed by the export receptor after mRNA binding facilitates its passage through the NPC [57]. Nuclear export factors act locally at the NPC by mediating recruitment of competent-mRNP onto the nuclear envelope and facilitating its recognition by the RNA Helicase DDX19 (Dbp5 in yeast) after translocation [58, 59]. At the NPC cytoplasmic face, the DEAD-box DDX19 triggers mRNP remodeling to displace the receptor and proteins from the mRNA [60]; DDX19 is activated by Gle1 (Gle1p in yeast) together with the small-molecule inositol hexakiphosphatase (InsP6) to stimulate RNA release to the translation machinery [61].

Another complex called TREX-2, which was first described in yeast [62], influences transcription and mRNA export [63]. TREX-2 is composed of Sac3, Thp1, Sem1, Sus1 and Cdc3 and regulates a surprisingly diverse number of chromatin-associated processes, including transcription and mRNA export, targeting of activated genes to NPCs, DNA replication, and genome stability [64–66]. In mammals, TREX-2 links transcription to the nuclear export by facilitating the transfer of mature mRNPs from the nuclear interior to NPCs [67, 68]. TREX-2 also promotes nuclear export of specific classes of mRNAs that may facilitate rapid changes in gene expression [9]. In summary, mRNA export involves TREX and TREX-2 complexes, which are formed by specific components.

## Unusual aspects of nucleocytoplasmic export in apicomplexan parasites

Comparative genomics analysis of proteins involved in RNA nucleocytoplasmic transport indicated that the basic components of the RanGTP-dependent RNA pathways are conserved across eukaryotes. On the other hand, several proteins involved in RanGTP-independent mRNA export pathways are not conserved, mainly in members of the supergroups Excavata and Chromalveolata, indicating lineage-specific innovations [11]. To provide a comprehensive review of the unusual aspects of nuclear export in apicomplexan parasites, we present a comparative analysis of transport factors that focuses on three representative species of the Apicomplexa (Additional file 1: Table S1). In general, results indicate that most of the components involved in the Ran-GTP pathway are conserved, though the numbers of components are reduced; however, only few components of mRNA export pathway are conserved in these representative species of apicomplexan parasites.

### RAN-GTP-dependent trafficking: Evidence of an atypical network

Studies on *T. gondii* using forward genetic approaches have led to the discovery of RCC1, a component of the RAN network that is involved in parasite virulence in vivo; deletion of the *T. gondii* divergent ortholog of RCC1 (TgRCC1) gene affects nuclear trafficking [69]. This was one of the first pieces of evidence of a divergent protein involved in *T. gondii* nuclear transport. Functional analysis of TgRCC1 indicates that although TgRCC1 is highly divergent, it nonetheless functions as a Guanine Exchange Factor (GEF) for Ran [70]. Furthermore, investigation has revealed that an atypical Ran network is required for parasite pathogenesis, highlighting that this pathway may represent a target for new therapeutic agents.

Although RCC1, Ran and several karyopherins involved in the protein transport that relies on the Ran-GTPase system are conserved in the Apicomplexa (Additional file 1: Table S1), RanGAP, an essential factor of the Ran-GTP gradient, does not appear to be encoded by the genome of any of three species analyzed herein, suggesting that either it is highly divergent or that the protein does not exist in these parasites. These results are in accordance with previous work in which the authors could not find RanGAP in any apicomplexan parasite genome [71], prompting the question of how *T. gondii* maintains a Ran gradient. Detailed analysis of nuclear trafficking is required to understand the mechanisms establishing the Ran gradient in apicomplexan parasites. However, the presence and characterized function of TgRCC1 suggest that such a gradient exists and that it may be established by unknown specific factors. Nuclear protein export/import pathways in apicomplexan parasites have been reviewed [71], and with the exception of the Ran GAP protein, apicomplexans appear to possess the key nuclear transport proteins of the Ran-GTP system, suggesting that these parasites may also harbor unique factors involved in this nucleocytoplasmic transport pathway.

Similar to protein transport, most of the exportins involved in the RNA export that relies on the Ran-GTP dependent pathway are found in the Apicomplexa. Although homologs of CRM1 and Exportin-t (t-RNA export) are present in apicomplexan genomes, a homolog for exportin-5 (snRNA export) is absent, suggesting that Exportin-t is most likely the main transporter of tRNAs and snRNAs in these parasites. In general, these results indicate that the Ran-GTP dependent export pathway for specific RNA species, such as rRNA, tRNA or snRNA, is likely conserved in these parasites.

### Ran-GTP-independent pathway: mRNA export may depend on highly divergent components

Unlike Opisthokonta lineages (such as yeasts and metazoans), several otherwise conserved key components of mRNA export are not found in the genomes of the Chromalveolata and Excavata lineages, including several species of parasites [11, 12]. Our bioinformatic analysis of Apicomplexa (Additional file 1: Table S1) corroborates previous work suggesting the presence of either highly divergent or unique components for mRNA export in these parasites. Figure 1b shows an overview of the few conserved components of mRNA export in the Apicomplexa, and the relevance of those findings are discussed below.

The major and specific mRNA complex (TREX) may not be conserved in the genomes of the three apicomplexan parasites we analyzed. These genomes contain only a homolog for UAP56 and lack a homolog for REF/Aly as well as for most THO complex components, with the exception of Tho2 (Additional file 1: Table S1). Similar to TREX, several homologs for components of the TREX-2 complex were not identified in these genomes, except for CDC31, which belongs to a complex that functions in gating transcription sites to the NPC and in maintaining genome stability [72–74]. Interestingly, EJC components that influence the fate of mRNA in eukaryotic cells are present in apicomplexan genomes, and this complex might have an important role in promoting the export of mRNA in these parasites. In summary, we speculate that apicomplexan parasites may possess specific pathways for identifying mRNA to be exported through gating active transcription sites at the proximity of the nuclear pore. However, we cannot exclude that the failure to identify several proteins might be due to the low conservation of sequences of export factors, precluding the identification of RNA nuclear export components via standard searches.

As mentioned above, genomic analyses revealed that Sub2/UAP56 is the only conserved component of TREX. Indeed, the conserved UAP56 homolog in *T. gondii* is crucial for mRNA export, given that conditional *uap56* knockout causes nuclear accumulation of poly(A) RNA [75]. Similarly, structural characterization of the *P. falciparum* UAP56 homolog revealed strong similarities to its human counterpart [76], indicating that its function may be conserved in apicomplexan parasites. Nonetheless, some *T. gondii* orthologs of factors specifically involved in the mRNA export pathway in the Opisthokonta, such as Gbp2, are essential proteins for parasite survival but are not crucial for mRNA export [75]. In addition, homology searches have been unsuccessful at identifying homolog of REF/Aly, another component of TREX, though a divergent ortholog of REF/Aly in *T. gondii* may fulfill the adaptor function of this protein [75].

In the absence of proteins crucial for mRNA export, it has been suggested that in apicomplexan parasites, the bulk of mRNA is transported via the CRM1-Ran pathway [11, 71]. However, using a CRISPR/Cas9 screen, it was demonstrated that neither TgCRM1 nor TgRan are essential for nuclear export of mRNA in *T. gondii*, indicating that a specific Ran-independent pathway is active in this parasite, as in other eukaryotes [75]. These data highlight the presence of divergent components and an atypical mRNA export pathway in *T. gondii* [73, 74].

The metazoan heterodimers TAP-p15 and yeast Mex67-Mtr2 are general mRNA export receptors in metazoans. Although a thorough search of apicomplexan genomes failed to identify a convincing homolog for Mex67/NXF1, an NXT1 homolog was identified in these genomes (Additional file 1: Table S1) [77]. It is puzzling that no sequences homologous to Mex67/NXF1 have been identified in apicomplexan genomes, as this protein is conserved from yeast to mammals and plays a central role in mRNA export [78]. It is likely that apicomplexans carry a functional homolog that is not recognizable by homology searches. Standard searches have failed to identify a homolog of the Mex67/NXT1 protein in trypanosomes, even though functional analysis of an RNA-binding protein has revealed the existence of a divergent functional homolog of Mex67 [15]. Interestingly, a protein bearing a C_2_H_2_ zinc finger domain (TgZNF2, TGME49_ 286710) was recently suggested to be a potential functional homolog Mex67/NXF1 [77]. This protein is conserved among apicomplexan parasites. When TgZNF2 is depleted in *T. gondii*, the parasites stop growing at the G1 phase of the cell cycle and accumulate poly(A) RNA in their nucleus [77]. Yeast Mex67 and mammalian NXF1 share little sequence identity, yet they have common recognizable motifs, such as leucine-rich repeats, NTF2-like domains and UBA-like domains. As none of these motifs are present in the TgZNF2 sequence, other proteins may have evolved to functionally replace Mex67/NXF1 in apicomplexan parasites. Strikingly, the *P. falciparum* TgZNF2 homolog complements *TgZNF2* depletion in *T. gondii*, indicating that the function of this protein may be conserved in these two parasites [77].

Proteomic analysis of TgZNF2 interacting partners has suggested that it interacts with spliceosome complexes. Therefore, TgZNF2 may interact with transcripts during the splicing process, a feature that is common among RNA export proteins in other eukaryotes [79]. Moreover, TgZNF2 interacts with TgNXT1, the homolog of NXT1 that is an important cofactor for Mex67/NXF1 and participates to the final step of RNA export through the nuclear pore [80]. TgZNF2 may therefore help shuttle mRNA outside of the nucleus through the NPC. In yeast and mammals, once the RNPs reach the cytoplasmic side of the NPC, the RNA helicase DDX19 stimulates mRNA release into the cytoplasm [81]. An homolog for DDX19 has been identified in apicomplexan genomes [77]; accordingly, proteins participating in mRNA release from the transport complex toward the cytoplasmic side of the nucleus may be conserved.

Searching for *T. gondii* NPC components has been successful using proteomic approaches [82]. Since most of these proteins do not share primary sequence homology with eukaryotic NUPs, as shown for *T. brucei* [83], structural conservation has enabled the identification of NUP homologs in *T. gondii*. Features of the *T. gondii* NPC components indicate conservation for the key features of the mRNP/NPC interaction [82]. For example, TgNup302 is a homolog of human Nup98/96, which interacts with TgRAE1 [82]. RAE1 is a shuttling transport factor that directly contributes to nuclear export of mRNA through its ability to anchor to a specific NUP98 motif at the NPC [84]. In yeasts and humans, this interaction is dependent on the presence of a GLEBS motif of Nup98/96 [84]. Interestingly, TgZNF2 also contains an active GLEBS motif that may be used as a platform for interaction with mRNA export proteins such as TgRAE1 [77].

Although they share structural similarities, *T. gondii* NPC components are rather large proteins compared with their eukaryotic counterparts. A CRISPR/Cas9 screen revealed that among the proteins identified as interacting with TgNup302, two are potentially involved in nuclear import [82]. These proteins may be localized at the cytosolic face of the NPC and serve as a platform for karyopherin interaction. Further studies may elucidate the specific roles of these proteins in nuclear transport. However, only one is conserved among apicomplexans, suggesting that protein nuclear import has potentially undergone divergence in this phylum.

## Conclusions

Studies on mRNA export in the Apicomplexa underline the differences between eukaryotic supergroups, such as the Opisthokonta and Chromalveolata. Although most studies to date have focused on *T. gondii*, the proteins discovered are conserved among different species and may have a role in mRNA export in apicomplexans (Additional file 1: Table S1). Further functional investigation in other apicomplexan species will validate the role of these proteins in this pathway. The main differences between *T. gondii* and other eukaryotes are the absence of two crucial proteins for mRNA export: REF/Aly, a TREX complex component, and the export receptor Mex67/NXF1. The discovery of highly divergent orthologs of these proteins (TgRRM1330 for REF/Aly and TgZNF2 for Mex67/NXF1) indicates that apicomplexan parasites may utilize a classical eukaryotic mRNA export pathway that comprises specific apicomplexan proteins.

The association of nascent mRNA with factors from the THO complex initiates the mRNA export during transcription in other eukaryotes. Thus, the apparent absence of the THO complex in apicomplexan genomes may be compensated for by other specific proteins to couple transcription and mRNA export. Along this line, the conserved EJC complex may serve as a platform for linking transcription, splicing and mRNP export since EJC travels with mRNA from its site of production to the cytoplasm [85]. Alternatively, apicomplexan parasites may employ a simplified pathway in which the spliceosome recruits mRNA export complexes via direct targeting of proteins such as TgZNF2.

The most puzzling question is the absence of a clear homolog of the RanGAP protein, which is indispensable for creating the Ran gradient that is required for protein export as well as for the recycling of many export factors [86]. Based on studies of members of the mRNA export pathway, we hypothesize that apicomplexan-specific proteins may fulfill this role.

Studies performed on trypanosomes also provide evidence of significant structural and functional differences in export factors with other eukaryotic lineages [13, 14]. Despite the high conservation of Sub2 in *T. cruzi* (TcSub2), its mode of action is not similar to the mammalian ortholog [87]. In accordance, the Mex67 receptor in *Trypanosoma brucei* (Tbmex67) contains a unique and essential N-terminal zinc finger motif and exhibits parasite-specific features [15]. In addition, there are exceptional structural dissimilarities between NPCs from animals/fungi and trypanosomes. In this case, kinetochores contains lineage-specific proteins and the lamina is apparently entirely distinct in trypanosomes [83]. In general, studies on nuclear biology of trypanosomatids have shown that key pathways present great adaptions. For example, there is considerable diversity in transcriptional control and genome segregation systems [88]. Therefore, parasites such as *Trypanosoma* or Apicomplexa, may also have a divergent mRNA export pathway that have undergone adaptations.

We herein discuss evidence that indicates the presence of lineage-specific proteins that may be involved in RNA export pathways in the Apicomplexa. These data highlight the need for large screens that allow the identification of key factors involved in these pathways. The CRISPR/Cas9 system has already proven its worth for genome-wide and more focused screens, and together with proteomic approaches, it will be useful for identifying candidates and other Apicomplexa-specific factors involved in nuclear mRNA export or other molecular pathways. In the case of trypanosome NPC, kinetochores and nuclear lamina, important progress in characterizing the molecular complexes composing these compartments were achieved by combining immunoprecipitation of protein complexes followed by proteomic analysis and reverse genetics. This detailed dissection of macromolecular complexes has provided a deeper appreciation of the evolutionary process between *Trypanosoma* and other eukaryotes [89, 90].

The proteins discovered in *T. gondii* are likely divergent functional homologs of key factors described in the Opisthokonta. Such structural modifications may constitute specific molecular adaptations that differentiate these proteins from their mammalian orthologs. Therefore, structure-based drug design can be useful to select drugs that could selectively inhibit the function of these proteins. These divergent proteins may represent attractive targets for drug development. Interestingly, such drugs may have an effect against a wide range of apicomplexan parasites if the target is conserved across the phylum. However, it is still necessary to explore in depth these apicomplexan-specific molecular mechanisms to provide a better understanding of their implication in the parasite biology.

## Additional file

Additional file 1: Table S1. Proteins involved in nuclear export in the three apicomplexan genomes analyzed. Identification number for proteins involved in nuclear import and export for the apicomplexan genomes, *Toxoplasma gondii*, *Plasmodium falciparum* and *Cryptosporidium* spp. are shown. Gene names for the human homologs are shown, and their corresponding annotation numbers are shown as references. When applicable, the name of the yeast homolog is indicated. Complexes with conserved components are highlighted in color. Statistics (E-value and % identity) are provided for each protein when compared to the human homolog. Conserved proteins are highlighted in bold. (*) indicates that these proteins were previously identified in the respective genomes in another study [77]. The *Toxoplasma gondii*, *Plasmodium falciparum* and *Cryptosporidium* spp. genomes were searched with the following keywords: “nuclear transport receptor”, “nuclear RNA export proteins”, “RNA export”, “importin alpha/beta”, “RNA-binding protein”, “regulator of nonsense transcripts”, “transportin”, “RAs-related nuclear protein/GTP-binding nuclear protein RAN”, “regulator of chromosome condensation”, “major exportin”, “exportins” and “THO complex”. Collected hits were translated into protein sequences, and domains were analyzed using Pfam [91]. To identify human homologs, the collected *T. gondii*, *P. falciparum* and *Cryptosporidium* spp. hits were searched against the *Homo sapiens* database using the Blastp tool from BLAST, and sequences with the lowest E-value were selected. *T. gondii*, *P. falciparum* and *Cryptosporidium* spp. homologs found were double-checked by searching the *T. gondii*, *P. falciparum* and *Cryptosporidium* spp. genome databases using the human homologs identified in the previous step as queries. Domains identified by Pfam were double-checked using Conserved Domain from BLAST. Using the above strategy, we were able to identify proteins at the protein family level, but the approach was not very accurate for identifying specific homologs. Thus, to identify specific homologs across relevant taxa, we systematically searched mammal, arthropod and apicomplexan databases using the Blastp tool from BLAST. (XLS 52 kb)

## Abbreviations

Cas9: CRISPR associated protein 9
CRISPR: Clustered Regular Interspaced Short Palindromic Repeats
FG: Phenylalanine (F) and glycine (G)
GDP: Guanosine 5′-diphosphate
GTP: Guanosine 5′-triphosphate
miRNA: Micro RNA
mRNA: Messenger RNA
mRNP: mRNA-protein complex
NPC: Nuclear pore complex
RCC1: Regulator of chromosome condensation 1
RNA: Ribonucleic acid
RNP: RNA-protein complex
rRNA: Ribosomal RNA
snRNA: Small nuclear RNA
THO: THO2 protein complex
TREX: Transcriptional export complex
tRNA: Transfer RNA
